# Rock magnetic and environmental magnetic data of lacustrine sediments from the Heqing basin

**DOI:** 10.1016/j.dib.2020.105107

**Published:** 2020-01-08

**Authors:** Xinwen Xu, Xiaoke Qiang, Hui Zhao, Chaofeng Fu

**Affiliations:** a Shaanxi Key Laboratory of Earth Surface System and Environmntal Carrying Capacity, Northwest University, Xi′an 710127, China; b State Key Laboratory of Loess and Quaternary Geology, Institute of Earth Environment, Chinese Academy of Sciences, Xi′an 710061, China; c Key Laboratory of Western Mineral Resources and Geological Engineering, Ministry of Education of China & Chang′an University, Xi′an 710054, China

**Keywords:** *The Heqing basin*, *Rock magnetism*, *Magnetic mineral dissolution*, *Indian monsoon*

## Abstract

This data article associated with the manuscript “Magnetic mineral dissolution recorded in a lacustrine sequence from the Heqing Basin, SW China, and its relationship with changes in the Indian monsoon”. Through detailed rock magnetic measurements, magnetic properties of the lacustrine sediments (magnetic mineralogy, their concentration and domain state) were clarified. Then, analyzing the relationship between magnetic property and paleoenvironmental proxies can reveal the paleoenvironmental implications of magnetic parameters of lacustrine sediments from the Heqing basin. Comparing paleoenvironmental proxies of lacustrine sediments from the Heqing basin with proxies associated with Indian monsoon recorded in Arabian Sea, and Bay of Bengal, can deepen our understanding about the characteristics of Indian Monsoon in the geological time.

**Table:** Specifications Table

Subject area	*Quaternary geology*
More specific subject area	*Rock magnetism and environment magnetism, paleoenvironment evolution*
Type of data	*Raw, table, figure*
How data was acquired	*Rock magnetic measurements were carried using Bartington Instruments MS2 magnetic susceptibility meter, MFK1-FA Kappabridge system, Model 3900 vibrating sample magnetometer.*
Data format	*Raw, filtered, analyzed, etc.*
Experimental factors	*Sample was dried below 30 °C.*
Experimental features	*Common rock magnetic measurement*
Data source location	*The Heqing drill core (26°33′43.1″N, 100°10′14.2″E) was obtained from the center of the Heqing Basin, southwestern China. All the measurements were carried out in Xi′an, China*
Data accessibility	*Rock magnetic and environment magnetic data in the article was deposited in Mendeley data Repository:*https://data.mendeley.com/datasets/rdvbmjx6tt/draft?a=184cb2a3-4780-4c53-8f2f-28f4361065c1.
Related research article	*Xu, X., Qiang, X., Zhao, H., Fu, C., 2019. Magnetic mineral dissolution recorded in a lacustrine sequence from the Heqing Basin, SW China, and its relationship with changes in the Indian monsoon. Journal of Asian Earth Sciences,*https://doi.org/10.1016/j.jseaes.2019.104081*.*

**Table:** 

**Value of the Data** • The dataset contain rock magnetic parameters of lacustrine sediments from the Heqing basin. The method can be applied to reveal the magnetic mineralogy, their concentration and domain state of materials like rock, sediment and soils, e.g.. • The lacustrine sediments were strong influenced by reductive dissolution of magnetic oxides. In environment magnetism research, the relationship between rock magnetic properties and magnetic mineral dissolution can be mirrored for the paleoenvironmental interpretation of the magnetic properties of lake and marine sediments. • The rock magnetic parameters (χ) revealed the characteristics of Indian Monsoon in SW China during the past 920 ka, and can deepen our understanding about the spatial distribution of characteristics of Indian Monsoon in different times.

## 1 Data

**Fig. 1:**
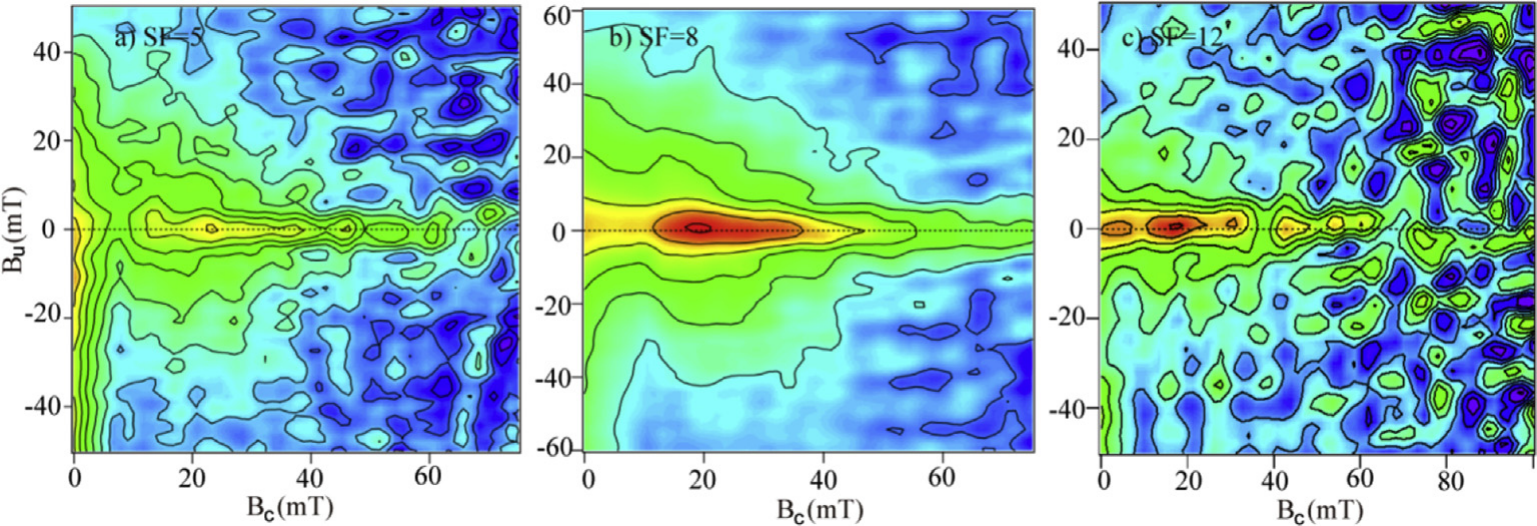
FORC diagrams for representative samples from the Heqing core. a-c) are high χ, intermediate χ and low χrepresentatively.

The dataset contains raw sequencing data obtained through different rock magnetic measurements. Rock magnetic parameter and its main controlling factors were listed in Table 1. Hyterisise parameters concluded saturation remanent magnetizatio (M_rs_), saturation magnetization (M_s_), coercivity of remanence (B_cr_) and coercive force (B_c_). FORC diagrams for representative samples were illustrated in Fig. 1. All these data were deposited in Mendeley data Repository linked with the related article.

**Table 1:** Magnetic parameters of the lacustrine sediments from the Heqing drill core.

magnetic parameter	Main control factor	quantity	Data type
χ	CM	877	raw
Temperature dependent χ	TM + DM	6	raw
Low temperature χ	TM	4	raw
Hyterisise parameters	TM + CM + DM	37	raw

χ: magnetic susceptibility; TM: type of the magnetic mineral; CM: concentration of magnetic mineral; DM: domain state of magnetic mineral.

## 2 Experimental design, materials, and methods

Magnetic susceptibility (χ) was measured using a Bartington Instruments MS2 magnetic susceptibility meter at a frequency of 465 Hz. Each sample was measured twice and the measurements were averaged. A sub-set of 41 representative samples were selected from the peaks and troughs of χ for detailed study. For the representative samples, magnetic susceptibility-versus-temperature curves (χ-T) were measured using a MFK1-FA Kappabridge system with a CS-3 high-temperature furnace. The temperature range was 40–700 °C with a heating rate of 11 °C per minute. The samples were heated and cooled in an argon atmosphere to minimize oxidation of sedimentary components. Low-temperature measurements were made using a MFK1-FA Kappabridge with a CS-L low-temperature furnace. The minimum temperature used was −194 °C. Hysteresis parameters and FORC diagrams were measured using a Model 3900 vibrating sample magnetometer (VSM), to a maximum applied field of 1 T. The ratio of saturation remanent magnetization to saturation magnetization (M_rs_/M_s_) against the ratio of coercivity of remanence to coercive force (B_cr_/B_c_) is plotted as Day-plot. All measurements were made at the Environmental Magnetism Laboratory in the Institute of Earth Environment, Chinese Academy of Sciences (Xi'an, China).

